# Molecular mechanisms of muscarinic receptors in mouse scleral fibroblasts: Prior to and after induction of experimental myopia with atropine treatment

**Published:** 2011-03-09

**Authors:** V.A. Barathi, Roger W. Beuerman

**Affiliations:** 1 Singapore Eye Research Institute, Singapore; 2 Department of Ophthalmology, Yong Loo Lin School of Medicine, National University of Health Sciences, Singapore; 3 Duke-NUS, SRP in Neurosciences and Behavioral Disorders, Singapore

## Abstract

**Purpose:**

To investigate the effect of atropine on the development of spectacle lens induced myopia in the mouse and to determine if the level of mRNAs for the muscarinic receptor subtypes (*M_1_* - *M_5_*) is affected by atropine treatment.

**Methods:**

Experimental myopia was developed in Balb/CJ (BJ) mice by placing −10 diopter spectacle lens on post-natal day 10 over the right eyes of 150 mice (n=10 in each group, 5 repetitions) for six weeks. After 2 weeks of lens wearing, the atropine group received a daily sub-conjunctival injection (10 µl) of 1% atropine sulfate and the saline group received daily 10 µl of 0.9% normal saline for 4 weeks. In addition, myopia was developed in C57BL/6 (B6) mice by placing −10 D spectacle lens on post-natal day 10 over the right eyes of 60 mice (n=10 in each group, 2 repetitions) for six weeks with and without atropine treatment. Refraction and axial length was measured at 2, 4, and 6 weeks after treatments. RT–PCR and northern blots were performed using specific primers for *M_1_*-*M_5_*, and products sequenced. Real-time PCR was used to quantify message levels.

**Results:**

Axial length of myopic eyes was 111% of their controls without atropine treatment and 103% of controls after atropine (p<0.01). Refraction shifted from myopic to emmetropic after atropine was administered in both pigmented and non-pigmented eyes. Corneal thickness, anterior chamber depth, corneal curvature and retinal thickness were not significantly different with and without atropine treatment (p=0.14). The lens thickness and vitreous chamber depth were significantly reduced after receiving atropine (p<0.05). Real-time PCR showed that message levels for *M_1_*, *M_3_*, and *M_4_* were upregulated in myopic sclera after atropine treatment, but *M_2_* and *M_5_* showed little change.

**Conclusions:**

The present study shows that 1% atropine reduces myopia progression in both pigmented and non-pigmented mice eyes. Axial length and vitreous chamber depth appear to be the main morphological parameters related to myopia. The results suggest that atropine may act on one or more muscarinic receptors to differentially regulate expression levels of specific receptors.

## Introduction

The cardinal optical characteristic of myopia is axial elongation of the posterior segment of the eye, which is due to scleral growth and remodeling [1-4]. Axial elongation is believed to be mediated by alterations in the connective tissues of the sclera [3,4], part of the collagenous outer tunic of the eye. To date, atropine (a pan-muscarinic antagonist) has proven to be a promising pharmacological agent that significantly reduced the progression of myopia in several clinical trials [5,6] including the ATOM (Atropine Treatment Of Myopia) study that was conducted in our center [7]. In a recent publication we demonstrated that the mouse and human scleral fibroblasts express all five types of muscarinic receptors [8]. However, it remains to be shown that atropine also halts the progression of myopia in the mouse model [9].

Atropine has been extensively tested in animal models. Suppression of deprivation myopia was studied with atropine treatment in tree shrews [10], chicks [11,12] and rhesus monkeys [13]. Since the mouse has recently been used as a new model [14,15] to study biologic aspects of myopia, the effects of atropine on experimental myopia are important to examine.

It is reasonable to expect that the mechanism of action of muscarinic receptor antagonists in inhibiting myopia progression to be consistent across species. Although atropine [16,17] and pirenzepine (specific muscarinic receptor 1 (M_1_) antagonist) [18] have both been shown to reduce myopia progression via slowing of axial elongation, the exact mechanism is still unknown. It was found that the M_1_ receptor does not exist in the chick sclera [19] such evidence implies that muscarinic antagonists which prevents the progression of myopia in the chick either work through another muscarinic receptor subtype or through non-specific or non-receptor mediated mechanisms. It is important to investigate the gene expression pattern during myopic development or progression may offer a productive avenue for future research.

The purpose of this study was to investigate the effect of atropine in a mouse model of experimental myopia and to determine if atropine prevents axial elongation after induction of spectacle lens induced myopia. The experimental myopia was developed in two different strains to determine the effect of spectacle lens induction in pigmented and non-pigmented eyes. Scleral fibroblast message levels for the muscarinic subtypes (*M_1_* - *M_5_*) were also analyzed in conjunction with atropine treatment during myopia progression.

## Methods

### Animals

Pregnant BJ mice (*Mus musculus*) and B6 mice were obtained from the animal holding unit of the National University of Singapore. Animals gave birth in our animal holding unit. Naive control animals were housed in groups of 6 while experimental animals were housed individually in standard mouse cages after 28 days of age at 25 °C on a schedule of 12:12 h of light on and off with mouse pellets and water available ad libidum. Approval was obtained from the SingHealth Institutional Animal Care and Use of Committee (IACUC) and all procedures performed in this study complied with the Association of Research in Vision and Ophthalmology (ARVO) Statement for the Use of Animals in Ophthalmology and Vision Research.

### Treatment protocols

The effects of atropine treatment were examined using spectacle lens-induced myopia and allocated to one of three groups: One group (n=10, 5 batch) received a daily 10 μl of 1% sub-conjunctival injections of atropine sulfate (ATG), and the other group (n=10, 5 batch) received daily 10 μl of 0.9% of sterile normal saline (NSG) as a vehicle, and the third group (n=10, 5 batch) was treated with spectacle lens alone to induce myopia. The right was used as a experimental and left eye was served as contra-lateral control in all groups. The same study was conducted in B6 mice (n=10, 2 batch in each group) with −10 diopter (D) to investigate the effect of strain difference with lens treatment. In all the remaining experiments, BJ treated mice samples were used.

Sub-conjunctival injections were administered to both eyes at the same time each day (approximately 9:00 AM) commencing on the 24th day (2 weeks after initiation of spectacle lens treatment). A compatible level of atropine was determined before the in vivo use in a tissue culture study with mouse scleral fibroblasts [8]. These concentrations (0.01% [147.929 µM], 0.1% [1479.29 µM], 0.5% [7396.45 µM], and 1% [14792.9 µM]) were then tested in vivo in a small pilot study. From our pilot study, we found that 0.5% and 1% atropine significantly reduced the elongation of axial length (data not shown). In this study, we are reporting results from the 1% atropine treatment. The injection (31.5 gauge needle) was performed under sterile conditions and microscopic control. For this procedure, mice were anaesthetized with 0.05–0.1 ml (IP) of a mixture of 0.2 ml 10% ketamine hydrochloride and 0.1 ml 2% xylazine hydrochloride, dissolved in 1.0 ml sterile saline. The eyes were examined daily and no infections were found. This treatment schedule continued for four weeks starting on post-natal day 24 continued until post-natal day 52. All measurements were taken at post-natal day 52, the equivalent of 6 weeks of spectacle lens wear.

### Refraction and axial length

Refractions and biometry measurements were recorded every week until the end of the study. Axial length, lens thickness, vitreous chamber depth and corneal diameter were measured with in vivo Optic Low Coherence Interferometry (OLCI-AcMaster). Refraction was measured by automated eccentric photorefractor. Details of the methods were previously described [14,15]. The corneal radius of curvature was measured in vivo by automated infrared photo-keratometry in B6 mice at all time points.

### Histological studies

Six eyes from each of the atropine and saline treated groups were fixed in freshly prepared 4% paraformaldehyde, pH 7.4 at room temperature (RT) for 24 h. Tissues were embedded in JB-4 plastic (Electron Microscopy Sciences, Hatfield, PA) overnight. Processed sections were stained with hematoxylin and eosin before coverslipping. Tissues were observed under light a microscope (LWDPL40xFPL-6, Olympus with a Nikon CoolpiX-995 Digital Camera (Olympus, Center Valley, PA) to record images) and sclera thickness measured using an ocular micrometer at 40× magnification. Measurements were obtained from three locations: just posterior to the limbus, at the equator and immediately lateral to the optic nerve head. For each location, three adjacent fields were measured and then averaged. Six sections from each eye were placed on a slide and the measurements were made from five individual mouse eye sections. The values used for each calculations represents the mean of ninety values for each locations.

### RNA Isolation and RT–PCR

Total RNA was isolated using TRIzol reagent (Invitrogen life technologies, Carlsbad, CA) in accordance with the manufacturer’s instructions [20]. Pairs of eyes were enucleated. The retina and choroid were stripped away from the sclera and immediately frozen in liquid nitrogen. Six different tissue samples were analyzed: 1. Atropine treated myopic sclera (AMS), 2. Control sclera from atropine treated mice (AMCS), 3. Normal saline treated myopic sclera (NSMS), 4. Normal saline treated control sclera (NSMCS), 5. Naive sclera (S) and 6. mouse cerebellum (C). Ten eyes were separately pooled and packed into aluminum foil and frozen as one sample. This experiment was repeated five times with different batches for all five tissues (n=10 in each group from 3 batch). A single pool of RNA from the cerebellum of three naive mice was used throughout the experiments as a control. In addition to the above mentioned tissues, myopic sclera (MS) and contra-lateral control sclera (CS) samples were also used for real-time PCR experiments (described below). PCR was performed as previously described [8].

### Northern blotting

Northern blot hybridizations were performed as previously described [21]. Briefly, 25 µg of total RNA was loaded in each lane, run on a 1% agarose gel, transferred to a positively charged nylon membrane, and hybridized to a fluorescein-labeled mouse M1 EcoRI enzyme digested insert cDNA clone.

### Real-Time Comparative PCR

Real-Time comparative PCR was performed in a 96-well microtiter plate format on an ABI PRISM 7700 Sequence Detection System (PE Applied Biosystems, Foster City, CA) equipped with a Sequence Detection System (SDS) software version 1.6.3. PCR was performed using 250 ng of cDNA of each sample. The primers/probes for muscarinic receptor subtypes; *M_1_*-*M_5_* were obtained from Taqman, Assays On Demand, PE Applied Biosystems. Primer sequences and conditions are presented in Table 1 Quantum RNA classic II 18S Internal Standard (Ambion, Austin, TX) was used as an endogenous control. The detailed method was previously described [14] and data was analyzed by comparative *C*_T_ (ΔΔ*C*_T_) method as previously described [22].

**Table 1:** Accession number of genes in the nucleotide sequence database (NCBI), sequences of used primer pairs and length of the amplified sequences

**Gene**	**Primer sequences**	**Size**	**Accession number**	**Percent homology**
*M1*	F: F: 5′ TCCCTCACATCCTCCGAAGGTG-3′	139 bp	NM_007698	99%
	R: R: 5′CTTTCTTGGTGGGCCTCTTGACTG-3′			
*M2*	F: F: 5′-CTGGAGCACAACAAGATCCAGAAT-3′	69 bp	NM_203491	100%
	R: R: 5′-CCCCCTGAACGCAGTTTTCAGT - 3′			
*M3*	F: F: 5′-GCAAGACCTCTGACACCAACT-3′	91 bp	NM_033269	100%
	R: R: 5′-AGCAAACCTCTTAGCCAGCG-3′			
*M4*	F: F: 5′-CGGCTACTGGCTCTGCTACGTCAA-3′	122 bp	NM_007699	100%
	R: R: 5′-CTGTGCCGATGTTCCGATACTGG-3′			
*M5*	F: F: 5′-TAGCATGGCTGGTCTCCTTCA-3′	76 bp	NM_205783	100%
	R: R: 5′-CGCTTCCCGACCAAGTACTG-3′			

### Data Analysis

Independent *t*-tests were used to analyze the means of the axial length, scleral thickness, and the refraction of the eyes. A difference at p value <0.05 was considered statistically significant. Data are reported as the mean±SD. The statistical significance of changes in *M_1_* - *M_5_* mRNA levels was analyzed using Student’s *t*-test. The Mann–Whitney U-test was used to determine differences between groups.

## Results

### Atropine treatment reduced axial elongation, lens thickness and vitreous chamber depth

**Figure 1:**
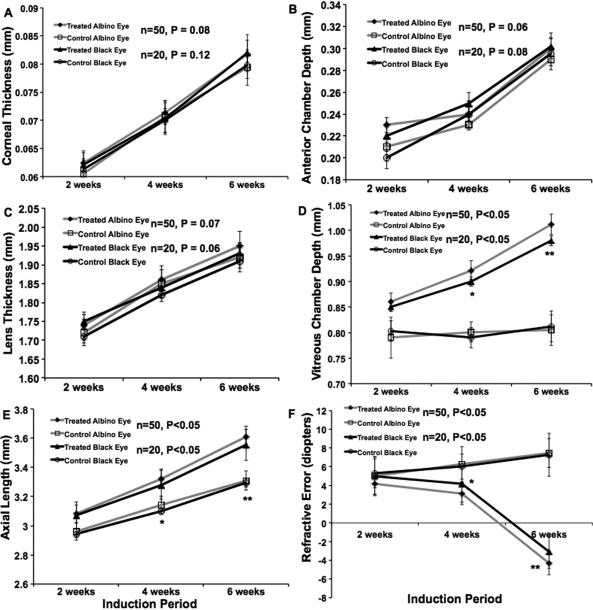
This graph represents the ocular biometry of Balb/CJ (BJ) and C57BL/6 (B6) mice with −10 D spectacle lens induced myopia. The lens was applied at post-natal day 10 (before eye opening). The ocular biometry measurements were measured using OLCI-AcMaster (in vivo: accuracy ±10 microns) and refraction (diopters) was measured by automated infrared photorefractor at 2 weeks, 4 weeks, and 6 weeks after induction of myopia. The BJ and B6 mice ocular biometry measurements (mm) were plotted against lens weraing period (weeks). The corneal thickness (**A**) and anterior chamber depth (**B**) was not significantly different when comparing the lens treated eyes against contra-lateral control eyes in both strains. The lens thickness (**C**), vitreous chamber depth (**D**), axial length (**E**), and refraction (**F**) were significant after 4 weeks and 6 weeks of induction in both strains. However when comparing the both strains, Balb/CJ mice eyes were more significant (n=50, p<0.01) at 6 weeks of minus lens wearing. Spectacle lens-induced myopia caused elongation of the globe and reduced hyperopia. Data was represented as mean±S.D, * represents significance level p<0.05 and ** represents significance level p<0.01.

**Figure 2:**
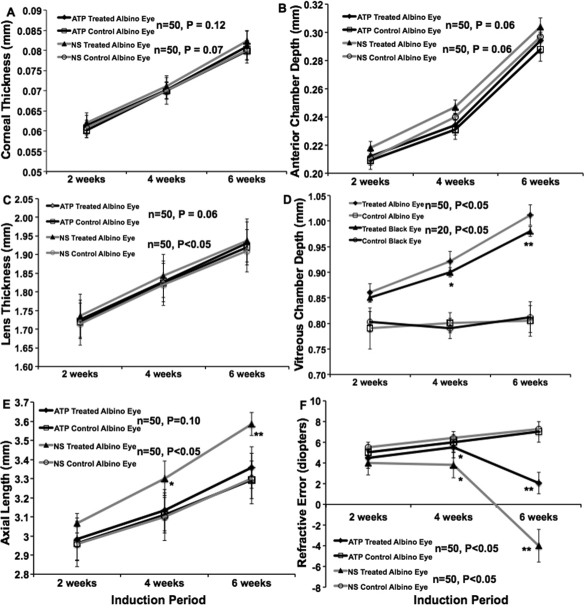
Atropine sulfate (pan muscarinic antagonist) and normal saline treated BJ mice ocular biometry measurements were plotted against induction period (weeks). One group was treated with sub-conjunctival injection of 1% atropine sulfate (pan muscarinic antagonist) and another group was treated with 0.9% normal saline for four weeks. The drug treatment was started after 2 weeks of minus lens wearing. Four weeks after drug treatment, refractive error and ocular biometry determined as before. The corneal thickness (**A**) and anterior chamber depth (**B**) was not significantly different with or without drug treatment. The lens thickness (**C**), vitreous chamber depth (**D**) and axial length (**E**) were significantly reduced after receiving atropine and there was no effect with saline treatment. The myopic eye received atropine sulfate was shifted from myopic to hyperopic when compared to saline treated myopic eye was till at myopic shift (**F**). There was no significant difference seen in the control eyes. Spectacle lens-induced myopic eye received 1% atropine reduced the myopia progression and not with saline treatment. This result confirms that 1% atropine reduces myopia progression in mice. Data was represented as mean±S.D, * represents significance level p<0.05 and ** represents significance level p<0.01.

**Figure 3:**
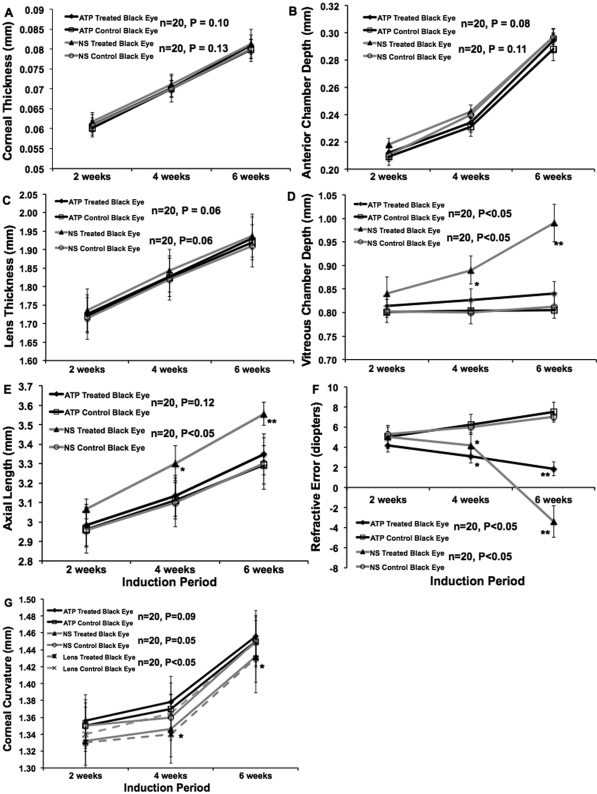
Atropine sulfate (pan muscarinic antagonist) and normal saline treated B6 mice ocular biometry measurements were plotted against induction period (weeks). One group was treated with sub-conjunctival injection of 1% atropine sulfate (pan muscarinic antagonist) and another group was treated with 0.9% normal saline for four weeks. The drug treatment was started after 2 weeks of minus lens wearing. Four weeks after drug treatment, refractive error and ocular biometry determined as before. The corneal thickness (**A**) and anterior chamber depth (**B**) was not significantly different with or without drug treatment. The lens thickness (**C**), vitreous chamber depth (**D**) and axial length (**E**) were significantly reduced after receiving atropine and there was no effect with saline treatment. The myopic eye received atropine sulfate was shifted from myopic to hyperopic when compared to saline treated myopic eye was till at myopic shift (**F**). There was no significant difference seen in the control eyes. The corneal curvature was determined by automated photokeratometry. The minus lens wearing eye’s corneal curvature was slightly flatter than the control and atropine treated eyes (**G**). This was significant at 4 and 6 weeks after induction of myopia. Spectacle lens-induced myopic eye received 1% atropine for 4 weeks was significantly reduced the axial elongation, lens thickness and vitreous chamber elongation however there was no effect with saline treatment. This result suggests that 1% atropine reduces myopia progression in mice and also no difference in strains. These results confirm that atropine is effective in reducing myopia progression in both pigmented and non-pigmented eyes. Data was represented as mean±S.D, *represents significance level p<0.05 and **represents significance level p<0.01.

Spectacle lens induction for a period of 42 days resulted in statistically significant in axial elongation, increased vitreous chamber depth of the treated eyes in BJ (p<0.01, n=50) and B6 mice (p<0.05, n=20) when compared to the contra-lateral control eyes. However the axial elongation, lens thickness, and vitreous chamber depth was appeared to be most significant in the BJ treated eyes as compared to the B6 treated eyes. There was no significant difference in corneal thickness and anterior camber depth between lens treated and contra-lateral eyes in both strains. (Figure 1A-E). Daily injections of the normal saline vehicle, into spectacle lens induced eyes did not alter the change in axial elongation, lens thickness and vitreous chamber depth in BJ (p<0.01, n=50) and B6 mice (p<0.05, n=20). Daily injections of atropine, at a concentration of 1%, blocked most of the elongation of axial length, lens thickness and the vitreous chamber depth associated with spectacle-lens induced myopia, but did not significantly affect the rate of axial elongation in contra-lateral control eyes. The other eye biometry measurements were not significantly different in treated or control eyes in non-pigmented and pigmented mice (Figure 2A-E and Figure 3A-E, respectively).

### Refractive error changes with atropine treatment

Eyes wearing −10 D lens in both non-pigmented and pigmented mice, the refraction was shifted from hyperopic to myopic (Figure 1F, p<0.01, n=50 and n=20, respectively) after 6 weeks of induction. Eyes wearing −10 D lens, treated with atropine sulfate for 4 weeks was shifted from myopic to hyperopic in both strains (Figure 2F, p<0.01, n=50 and 3F, p<0.01, n=20, respectively) were significant when compared to normal saline treatment. Whereas the minus lens wearing eye, treated with saline for 4 weeks was still showed a myopic shift in both strains (Figure 2F and Figure 3F, respectively). This result indicates that the atropine treatment reduces the progression of myopia and not the saline vehicle treatment. There was no significant difference seen in the contra-lateral control eyes in both strains (Figure 2F and Figure 3F, respectively). In B6 mice, the minus lens wearing eye’s corneal curvature was slightly flatter than the control and atropine treated eyes (Figure 3G). This was significant at 4 and 6 weeks after induction of myopia (p<0.05, n=20).

### Comparative analysis by real time PCR

Data from three different comparisons was analyzed. Seven different tissues were used for quantification of muscarinic receptor gene expression: 1. MS (minus lens wearing), 2. CS (no lens), 3. AMS (minus lens with atropine), 4. AMCS (no lens with atropine) 5. NSMS (minus lens with saline), 6. NSMCS (no lens with saline), 7. S (Naive sclera), 8. Mouse Brain cerebellum was used as positive control and 18s rRNA was used as endogenous internal control for analysis.

### Gene expression of myopic and atropine treated myopic sclera

**Figure 4:**
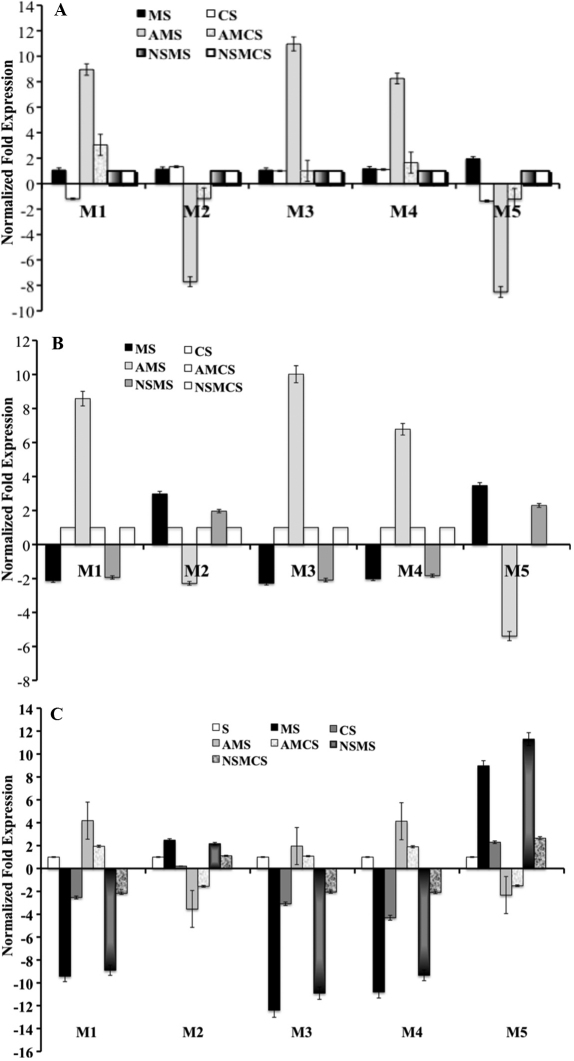
Relative gene expression of muscarinic receptor 1-5 to the corresponding atropine treated mRNA level with and without myopia. **A**: Bar graph depicting the relative gene expression of muscarinic receptor 1–5 of myopic (MS) and atropine treated myopic (AMS) sclera ∆C_T_ values to the corresponding normal saline treated (NSMS) sclera mRNA level after normalization with 18S rRNA internal standard. Similar analysis method was applied to the control group (NSMCS). The mRNA level of *M_1_*, *M_3_*, and *M_4_* after atropine treatment in the experimental myopic sclera (AMS) was upregulated and some change observed in the atropine treated control (AMCS) whereas down regulated during myopia (MS) and after receiving saline (NSMS). The mRNA levels of *M_2_* and *M_5_* after induction of myopia were upregulated and reversed (down-regulated) in atropine treated group. Data was represented as mean±SD **B**: Bar graph depicting the relative gene expression of muscarinic receptor 1–5 of myopic (MS), atropine treated myopic (AMS) and normal saline treated myopic (NSMS) sclera ∆C_T_ values to the corresponding their own contra-lateral control mRNA level (CS, AMCS, and NSMCS, respectively) after normalization with 18S rRNA internal standard. The mRNA levels of *M_1_*, *M_3_*, and *M_4_* after induction of myopia and treated with normal saline were down regulated and reversed (upregulated) in atropine treated group. The mRNA levels of *M_2_* and *M_5_* after induction of myopia and treated with normal saline were upregulated and reversed down-regulated in the atropine treated sclera. Data was represented as mean±SD **C**: Bar graph depicting the relative gene expression of muscarinic receptor 1–5 of cerebellum, myopic (MS), atropine treated myopic (AMS), normal saline treated myopic (NSMS), contra-lateral control (CS), atropine treated control (AMCS) and normal saline treated control (NSMCS) sclera ∆C_T_ values to the corresponding the naive sclera (S) mRNA level after normalization with 18S rRNA internal standard. The mRNA levels of *M_1_*, *M_3_*, and *M_4_* after induction of myopia and treated with normal saline were down regulated and reversed (upregulated) in atropine treated group. The mRNA levels of *M_2_* and *M_5_* after induction of myopia and treated with normal saline were upregulated and opposite in the atropine treated group. Data was represented as mean±SD.

Initially, C_T_ values for *M_1_*-*M_5_* in MS and AMS were compared against NSMS of muscarinic receptors gene C_T_ values after normalizing with 18s rRNA. Similar analysis method was applied to the control group. *M_1_*, *M_3_*, and *M_4_* mRNA levels were upregulated in the AMS and AMCS. In contrast, *M_2_* and *M_5_* mRNA levels were down regulated in the AMS and AMCS (Figure 4A). There were no changes in the MS and CS *M_1_*-*M_5_* mRNA levels.

Normalized fold change expression of *M_1_*-*M_5_* in AMS as compared with NSMS were 11, −8, 9, 8.5, −9.2 fold change (p<0.01, n=5 experimental repeats), respectively. The effects of atropine on message levels for *M_1_*, *M_3_*, and *M_4_* were greater in experimental myopic eyes than the contra-lateral control (non-myopic) eyes. Similarly atropine effect on *M_2_* and *M_5_* was much greater in experimental myopic eyes than control eyes. It was reported that atropine reduces the axial length even in the naive eyes [23,24] however the effect was much higher in the spectacle lens wearing eyes.

### Gene expression of myopic, atropine treated, saline treated compared with contra-lateral control sclera

Message levels for the muscarinic receptors from MS was compared against the CS from the same animal using the gene C_T_ value after normalizing with 18s rRNA. Normalized fold change expression of *M_1_* (−2.12 fold), *M_3_* (−2.27 fold), and *M_4_* (−2.02) mRNA levels were down-regulated in the MS and NSMS (−1.93 fold, −2.09 fold and −1.83 fold, respectively; p<0.01) whereas *M_1_*, M_3_, and *M_4_* mRNA levels were upregulated after receiving atropine (10.58 fold, 5.01 fold and 6.78 fold, respectively; p<0.01). In contrast, *M_2_* (2.98 fold) and *M_5_* (3.47 fold) mRNA levels were upregulated in the MS and NSMS (1.96 fold, 2.3 fold respectively; p<0.01). This was opposite in the AMS (*M_2_*: −2.28 and *M_5_*: −5.39 fold; Figure 4B).

Analysis showed that atropine effect on *M_1_* was much greater in MS as compared to their control after being normalized with 18s rRNA than on *M_4_* and *M_3_* (125%, 115%, and 112%, respectively; p<0.01). Similarly atropine effect on *M_5_* was much greater in MS than it was on *M_2_* (116%. and 101%, respectively; p<0.01). Our results showed that M_1_, *M_3_*, and *M_4_* levels of scleral mRNA reduction, which led to the suppression of excessive axial elongation while *M_2_* and *M_5_* showed little change.

### Gene expression of experimental and control sclera against naive sclera

Experimental, control scleral, and mouse cerebellum muscarinic receptor subtypes C_T_ values were compared against naive sclera of *M_1_*-*M_5_* C_T_ values after being normalized with 18s rRNA. *M_1_* mRNA levels were down regulated (−9.52 fold) after the induction of myopia and treated with normal saline (−9.22 fold) and this expression pattern was reversed with atropine treatment (4.34 fold). The mRNA level of *M_3_* and *M_4_* showed a similar expression trend to that of *M_1_*. In contrast, *M_2_* and *M_5_* mRNA levels were upregulated in MS and NSMS whereas it was highly down regulated in the AMS (Figure 4C). The atropine effect on *M_1_*, *M_2_*, and *M_4_* was much greater in experimental myopic eyes when compared to naive sclera.

In all set of data, MS and NSMS gene expression patterns for *M_1_*-*M_5_* was similar but not the exact values as expected. The morphological and structural changes of sclera in both treatments were not different. Moreover, the mRNA levels were slightly different in these 2 groups of sclera; this may be due to some external stress received during saline injection or other factors involved.

### Northern blot analysis

**Figure 5:**
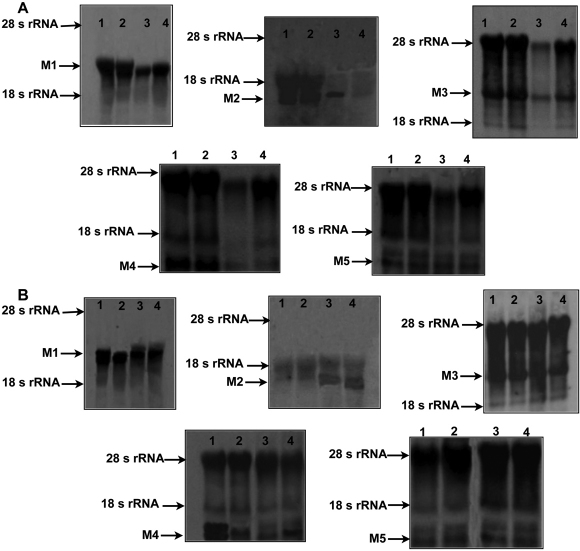
Northern blot analysis. Northern blot of *M_1_*-*M_5_* mRNA expression in 6 weeks minus lens treated sclera (**A**) and with atropine or saline treated sclera (**B**). Total RNA (25 µg) was loaded in each lane, run on a 1% agarose gel, transferred to a positively charged nylon membrane, and hybridized to a fluorescein-labeled mouse M1 EcoRI enzyme digested insert cDNA clone. In the upper panel the sizes of 28S (4.7 kb), 18S (1.9 kb) rRNA and *M_1_*-*M_5_* (2.6 kb, 1.8 kb, 3.2 kb, 1.6 kb and 3.5 kb, respectively) are indicated to the left. **A**: Lane 1: Mouse brain cerebellum (positive control), lane 2: minus lens treated myopic sclera, lane 3: minus lens control sclera, lane 4: naive sclera. **B**: Lane 1: atropine treated myopic sclera, lane 2: atropine treated control sclera, lane 3: saline treated myopic sclera, lane 4: saline treated control sclera.

To corroborate the positive results obtained from PCR, northern blots, a standard method for detection of mRNA levels, were performed. At the same time northern blots also provide a direct comparison of message abundance between samples on a single membrane. Northern blot analysis confirmed the same pattern of gene expression for all five *M_1_*-*M_5_* in the minus lens treated sclera (Figure 5A), lens with atropine or normal saline treated experimental sclera and control sclera (Figure 5B) as like Real-Time PCR results.

### Expression of muscarinic receptor subtypes

**Figure 6:**
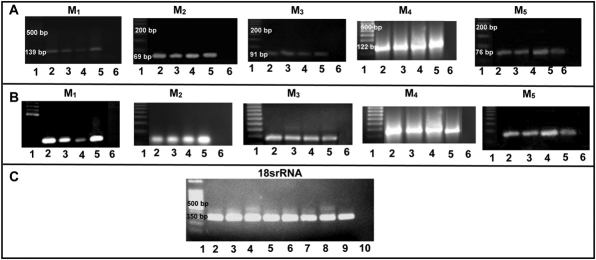
Gene expression of muscarinic receptor 1-5 prior to and after induction of experimental myopia with atropine treatment. RT–PCR results for *M_1_*-*M_5_* gene expression in 6 weeks minus lens treated sclera (**A**) and with atropine or saline treated sclera (**B**). **A**: Lane 1: DNA ladder, lane 2: Mouse brain cerebellum (positive control), lane 3: minus lens treated myopic sclera, lane 4: minus lens control sclera, lane 5: naive sclera, lane 6: water (negative control). **B**: Lane 1: DNA ladder, lane 2: atropine treated myopic sclera, lane 3: atropine treated control sclera, lane 4: saline treated myopic sclera, lane 5: saline treated control sclera, 6: water (negative control). **C**: 18s rRNA was also loaded in parallel to detect the DNA contamination for all samples used.

RT–PCR was performed on sclera from mice treated with minus lens, receiving atropine and normal saline in conjunction with development of myopia, contra-lateral controls, and naive sclera at the end of each experiment. It was determined that *M_1_*-*M_5_* was differentially expressed in mice treated with minus lens with and without drug treatment and, contra-lateral control sclera (Figure 6A,B, respectively). In preliminary studies it was found that the pigment epithelium was often closely adherent to the sclera. Therefore, a procedure was developed to quickly remove the pigment epithelium, which was checked by histological evaluation of sections from different regions of the sclera. Therefore the PCR results were considered to represent only the sclera fibroblasts [8].

### Atropine treatment reversed scleral thinning

**Figure 7:**
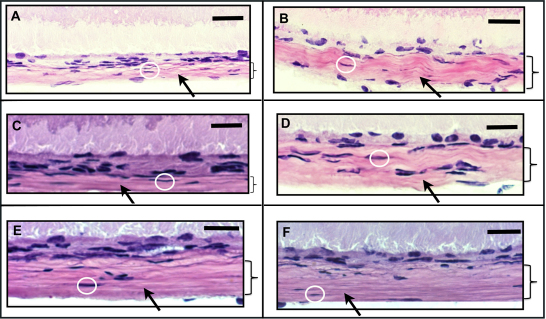
Light micrographs of plastic-embedded mouse posterior sclera. Photomicrograph of posterior scleral thickness of spectacle lens treated (**A**) and control sclera (**B**). Normal saline treated myopic (**C**) and contra-lateral control eyes (**D**, n=6 eyes, 6 sections from each eye), 40× original magnification. There was no effect of normal saline on the posterior scleral thickness of myopic sclera. Lens treatment with and without normal saline reduced the sclera thickness which shows that scleral thinning occurred during myopia development and there was no effect of normal saline treatment in the myopia progression. Atropine treated myopic (**E**) and atropine treated contra-lateral control eyes (**F**, n=6 eyes, 6 sections from each eye), 40× original magnification. Atropine sulfate was increased the posterior scleral thickness which shows that 1% atropine was effective in the myopia progression in mouse. Arrow indicates the sclera and box indicates the thickness. Scale bar=50 μm. Black arrows indicate the sclera and white circles show the scleral fibroblasts.

As known from previous observations in the normal mammalian eye, it was found in the mouse that scleral thickness increased from anterior to posterior in naive and control eyes. Scleral thickness was measured from photomicrographs using the calibrated stage micrometer (accuracy ±7.0 µm) for as well as by magnified video imaging using trans-illuminated globes (accuracy ±4.0 µm). As shown in Table 2 (all at a level of p<0.05, n=6 in each group from 3 batch), spectacle lens induced myopia (Figure 7A) combined with daily injections of normal saline resulted in reductions in scleral thickness at equatorial and posterior locations (Figure 7C) when compared to aged-matched untreated eyes (Figure 7B) and with only normal saline without lens treatment (Figure 7D). Spectacle lens induced myopia with daily injection of atropine sulfate, at a concentration of 1%, blocked the posterior scleral thinning (Figure 7E), this was closely similar to their fellow eyes without lens treatment (Figure 7F).

**Table 2:** Spectacle lens, atropine, and saline treated group mice scleral thickness versus contra-lateral control scleral thickness.

**Dimensions (µm)**	**Ex (Atr)**	**Con (Atr)**	**Ex (NS)**	**Con (NS)**	**Ex**	**Con**	**Naive**	**Age (Days)**
Anterior Sclera	16.7±0.06	16.7±0.10	16.4±0.02	16.9±0.07	16.5±0.06	16.6±0.08	16.9±0.10	52
Equator Sclera	32.3±0.08	31.7±0.08	22.6±0.10	30.8±0.13	22.2±0.09	31.0±0.10	31.8±0.12	52
Posterior Sclera	70.5±0.05	71.2±0.03	53.9±0.05	70.9±0.09	53.2±0.08	71.1±0.09	71.7±0.11	52

Ex (Atr)=atropine treated myopic eye, Con (Atr)=atropine treated control eye, Ex (NS)=normal saline treated myopic eye, Con (NS)=normal saline treated control eye, Ex=myopia induced eye, Con=control eye. Values are represented as mean ±S.D, significance at p<0.05, n=10 for each group.

## Discussion

In this study, BJ mouse was used to induce experimental myopia as per prior established method [14]. Experimental myopia was achieved for our study even though we had used albino strain which suggests that visual cortex is not important to control the excessive expansion of the posterior segment of the eye or refractive error changes in mouse. Our results attained were similar to past reported studies of different species which are elaborated below [25-28]. Deprivation of various parts of the visual field produces myopia and elongation even in animals with optic nerve severed or those affected by degraded retinal image (even if it is in one sector of the eye) impling that accommodation is not involved. The mechanism by which the nervous system influences eye growth appears to be different even in closely related primate species which exhibit different responses to form deprivation conditions, suggesting differing mechanisms of eye growth control. Paralysis of the ciliary muscle or optic nerve section does not prevent the development of myopia in the rhesus macaque, indicating that in this species the axial growth is controlled by the retina. These results suggest that some factor in part of the retina can influence the growth of the sclera or the level of the scleral ocular elongation results from local growth, because we found increases in scleral fibroblasts cell proliferation in the induced eye (data not shown). It implies that the control of eye growth by vision seems to take place in local regions of the eye.

B6 mice were also used in our study to determine the effect of atropine in pigmented eyes with myopia. The results confirm that both pigmented and non-pigmented eyes were influenced by the minus lens treatment. Elongation of axial length and vitreous chamber depth appeared to be the main morphological parameters related to myopia in both strains. Moreover the axial length elongation was most significant in BJ mice when compared to B6 mice which indicates that the eye growth was higher in the BJ mice with age or with minus lens treatment.

Atropine was injected sub-conjunctively through the upper bulbar conjunctiva, a route that optimizes scleral exposure to the drug to determine the muscarinic receptor gene expression pattern during myopia development. It has been proposed that muscarinic antagonists might produce their effect by acting directly on scleral muscarinic receptors; however, that hypothesis is still being investigated [29]. We have confirmed that atropine effectively reduced axial elongation in mouse eyes undergoing spectacle lens treatment to induce myopia. Atropine did not impose significant changes in the axial length of control eyes, which received clear visual input. This finding is in agreement with previous studies that investigated the effects of atropine on FDM in chicks [11,12], in tree shrews [30] and in monkeys [13,31,32]. Atropine has been shown to effectively prevent the progression of human juvenile myopia [33,34]. In mouse, the atropine reduces the eye growth at both low (data not shown) and high doses (this study) but the role of muscarinic receptors remains unclear. The application of anti-muscarinic drugs were able to change collagen structure and production of scleral extracellular proteins during myopia development in animal models [35,36]. This tend to suggest that investigation of scleral remodeling by atropine in the mouse model may be a good strategy to discover other anti-myopiagenic drugs for humans.

The mouse sclera, a connective tissue consisting of fibroblasts embedded in an extracellular matrix (ECM) of largely collagen Type I and proteoglycans, defines the shape and axial length of the eye. Because both biochemical analysis and histological analysis showed, significant changes only occur within the posterior region of sclera in chick model [37,38] but without evidence in the mouse model, the histological study was performed on the whole sclera. From our findings, in anterior and equator sclera of experimental eye, there was no obvious change when compared to that of control eyes. As such, the posterior sclera was thinner than that of control eye. These results are consistent with those from the chick [39]. The fibrous sclera responds in the same manner across species (birds, mammals, and human) in response to hyperopic defocus or form deprivation stimuli, thinning in experimental eyes. The change in axial length could potentially be attributed to changes in posteriorly located ocular tissues, such as vitreous, retina or sclera. These ambiguous findings suggest that in the chick, atropine may work via other mechanism to inhibit myopia development because it was reported that the chick does not possess a functional M_1_ receptor [19]. It would be important to determine if muscarinic antagonists interact with other pathways implicated in the regulation of ocular growth.

*M_1_*, *M_3_*, and *M_4_* gene expression levels were upregulated in sclera of the experimental eyes following atropine treatment. After the treatment, myopic sclera produced less message for *M_2_* and *M_5_* and more in saline treated myopic sclera. This is the first study that reports differential regulation and expression of mRNA levels following chronic administration of atropine in experimentally induced myopic mice. Comparison of these results with those of previous studies on rat brain [40], chick cerebral neurons [41], and rat cortex [42,43], indicates that differential regulation of *M_1_*-*M_5_*; *M_1_*, *M_3_*, and *M_4_* were upregulated whereas *M_2_* and *M_5_* were down regulated in response to chronic administration of atropine. Thus, at both the protein and message level, these two receptor systems seem to be about equal in abundance in cortex.

In rat cerebellar cells, *M_2_* and *M_3_* mRNAs were down regulated with carbachol treatment [44] however, antagonist induced upregulation of *M_2_* and *M_3_* mRNA level [45]. In rabbit tracheal smooth muscle, *M_3_* was upregulated following atropine treatment [46]. In guinea pig posterior sclera, *M_1_* and *M_4_* mRNA levels were increased after induction of form-deprived myopia [47]. It is clear that the regulation of *M_1_*-*M_5_* subtypes varies between species and different tissues or cell types. Differential expression of muscarinic receptors suggests that different routes of administration and concentrations of atropine would have influence on the mRNA levels.

In conclusion, we have confirmed that atropine effectively reduces progression of myopia in both pigmented and non-pigmented mice. Muscarinic receptor antagonists most certainly seem to be promising drugs to inhibit human myopia. Additional knowledge on the actions of the drug, its toxicity and the mechanism are required. We have determined the mechanism of atropine at the gene expression level however there are avenues to be explored on the molecular and protein level. In this regard, the study of changing patterns of gene expression within and among species during emmetropization and myopic progression may offer a productive avenue for future research. Elucidating deficient steps in the regulatory pathway would mark significant advance given myopia's tremendous impact. No data are available on the systemic toxic effect in the long term usage of atropine. By studying the atropine treatment on muscarinic knock out mouse model would help us in understanding the effect of long-term usage of atropine or specific muscarinic blockers in human subjects and further investigations that need to be conducted with new therapeutic agents to treat myopia with this new model.

## References

[1] Funata M, Tokoro T (1990). Scleral change in experimentally myopic monkeys. Graefes Arch Clin Exp Ophthalmol.

[2] Norton TT (1990). Experimental myopia in tree shrews.. Ciba Found Symp.

[3] McBrien NA, Gentle A (2001). The role of visual information in the control of scleral matrix biology in myopia.. Curr Eye Res.

[4] McBrien NA, Gentle A (2003). Role of the sclera in the development and pathological complications of myopia.. Prog Retin Eye Res.

[5] Shih YF, Chen CH, Chou AC (1999). Effects of different concentrations of atropine on controlling myopia in myopic children.. J Ocul Pharmacol Ther.

[6] Chou AC, Shih YF, Ho TC, Lin LL (1997). The effectiveness of 0.5% atropine in controlling high myopia in children.. J Ocul Pharmacol Ther.

[7] Chua WH, Balakrishnan V, Chan YH, Tong L, Ling Y, Quah BL, Tan DT (2006). Atropine for the Treatment of Childhood Myopia.. Ophthalmology.

[8] Barathi VA, Weon SR, Beuerman RW (2009). Expression of muscarinic receptors inhuman and mouse sclera and their role in the regulation of sclera fibroblast proliferation.. Mol Vis.

[9] Barathi VA, Beuerman RW (2006). Muscarinic mechanisms in a mouse model of myopia.. Ophthalmic Physiol Opt.

[10] Mckanna JA, Casagrande VA (1985). Chronic cycloplegia prevents lid-suture myopia in tree shrews.. Invest Ophthalmol Vis Sci.

[11] Schwahn HN, Kaymak H, Schaeffel F (2000). Effects of atropine on refractive development, dopamine release, and slow retinal potentials in the chick.. Vis Neurosci.

[12] Wildsoet CG, McBrien NA, Clark IQ (1994). Atropine inhibition of lens-induced effects in chick: evidence for similar mechanisms underlying form deprivation and lens induced myopia.. Invest Ophthalmol Vis Sci.

[13] Tigges M, Iuvone PM, Fernandes A, Sugrue MF, Mallorga PJ, Laties AM, Stone RA (1999). Effects of muscarinic cholinergic receptor antagonists on postnatal eye growth of rhesus monkeys.. Optom Vis Sci.

[14] Barathi VA, Boopathi VG, Yap ETH, Beuerman RW (2008). Two models of experimental myopia in mouse.. Vision Res.

[15] Schaeffel F, Burkhardt E, Howland HC, Williams RW (2004). Measurement of refractive state and deprivation myopia in two strains of mice.. Optom Vis Sci.

[16] Saw SM, Gazzard G, Au Eong KG, Tan DT (2002). Myopia: attempts to arrest progression.. Br J Ophthalmol.

[17] Tong L, Huang XL, Koh AL, Zhang X, Tan DT, Chua WH (2009). Atropine for the treatment of childhood myopia: effect on myopia progression after cessation of atropine.. Ophthalmology.

[18] Tan DT, Lam DS, Chua WH, Shu-Ping DF, Crockett RS, Asian Pirenzepine Study Group (2005). One-year multicenter, doublemasked, placebo-controlled, parallel safety and efficacy study of 2% pirenzepine ophthalmic gel in children with myopia.. Ophthalmol.

[19] Yin GC, Gentle A, McBrien NA (2004). Muscarinic antagonist control of myopia: a molecular search for the M1 receptor in chick.. Mol Vis.

[20] Chomczynski P, Sacchi N (1987). Single-step method of RNA isolation by acid guanidinium thiocyanate-phenol-chloroform extraction.. Anal Biochem.

[21] Liu Y, Jovanovic B, Pins M, Lee C, Bergan RC (2002). Over expression of endoglin in human prostate cancer suppresses cell detachment, migration and invasion.. Oncogene.

[22] Brink N, Szamel M, Young AR, Wittern KP, Bergemann J (2000). Comparative quantification of IL-1, IL-10, Il-10r, TNF and IL-7 mRNA levels in UV-irradiated human skin in vivo.. Inflamm Res.

[23] Barathi VA, Beuerman RW, Schaeffel F (2009). Effects of unilateral topical atropine on binocular pupil responses and eye growth in mice.. Vision Res.

[24] Schaeffel F, Burkhardt E (2005). Pupillographic evaluation of the time course of atropine effects in the mouse eye.. Optom Vis Sci.

[25] Duncan G, Collison DJ (2003). Role of the non-neuronal cholinergic system in the eye: a review.. Life Sci.

[26] Raviola E, Wiesel TN (1990). Neural control of eye growth and experimental myopia in primates.. Ciba Found Symp.

[27] Sivak JG, Barrie DL, Callender MG, Doughty MJ, Seltner RL, West JA (1990). Optical causes of experimental myopia.. Ciba Found Symp.

[28] Wallman J (1990). Retinal influences on sclera underlie visual deprivation myopia.. Ciba Found Symp.

[29] Lawrence MS, Azar DT (2002). Myopia and models and mechanisms of refractive error control.. Ophthalmol Clin North Am.

[30] Cottriall CL, McBrien NA (1996). The M (1) muscarinic antagonist pirenzepine reduces myopia and eye enlargement in the tree shrew.. Invest Ophthalmol Vis Sci.

[31] Raviola E, Wiesel TN (1985). An animal model of myopia.. N Engl J Med.

[32] Ashkenazi A, Ramachandran J, Capon DJ (1989). Acetylcholine analogue stimulates DNA synthesis in brain-derived cells via specific muscarinic receptor subtypes.. Nature.

[33] Bedrossian RH The effect of atropine on myopia. Am J Ophthalmol 1979 86 713 7 545205 10.1016/s0161-6420(79)35455-0 545205

[34] Brenner RL (1985). Further observations on use of atropine in the treatment of myopia.. Ann Ophthalmol.

[35] Lind GJ, Chew SJ, Marzani D, Wallman J (1998). Muscarinic acetylcholine receptor antagonists inhibit chick scleral chondrocytes.. Invest Ophthalmol Vis Sci.

[36] McBrien NA, Metlapally R, Jobling AL, Gentle A (2006). Expression of collagen-binding integrin receptors in the mammalian sclera and their regulation during the development of myopia.. Invest Ophthalmol Vis Sci.

[37] Kusakari T, Sato T, Tokoro T (1997). Regional scleral changes in form-deprivation myopia in chicks.. Exp Eye Res.

[38] Rada JA, Mathews AL, Brenza H (1994). Regional proteoglycan synthesis in the sclera of experimentally myopic chicks.. Exp Eye Res.

[39] Gottlieb MD, Joshi HB, Nickla DL (1990). Scleral changes in chicks with form-deprivation myopia.. Curr Eye Res.

[40] Wall SJ, Yasuda RP, Li M, Ciesla W, Wolfe BB (1992). Differential regulation of subtypes m1-m5 of muscarinic receptors in forebrain by chronic atropine administration.. J Pharmacol Exp Ther.

[41] Siman RG, Klein WL (1979). Cholinergic activity regulates muscarinic receptors in central nervous system cultures.. Proc Natl Acad Sci USA.

[42] McKinney M, Robbins M (1992). Chronic atropine administration up-regulates rat cortical muscarinic m1 receptor mRNA molecules: assessment with the RT/PCR.. Brain Res Mol Brain Res.

[43] Waelbroeck M, Tastenoy M, Camus J, Christophe J (1990). Binding of selective antagonists to four muscarinic receptors (M1 to M4) in rat forebrain.. Mol Pharmacol.

[44] Fukamauchi F, Saunders PA, Hough C, Chuang DM (1993). Agonist-induced down-regulation and antagonist-induced up-regulation of m2- and m3-muscarinic acetylcholine receptor mRNA and protein in cultured cerebellar granule cells.. Mol Pharmacol.

[45] Fukamauchi F, Hough C, Chuang DM (1991). Expression and agonist-induced down-regulation of mRNAs of m2- and m3-muscarinic acetylcholine receptors in cultured cerebellar granule cells.. J Neurochem.

[46] Witt-Enderby PA, Yamamura HL, Halonen M, Lai J, Palmer JD, Bloom JW (1995). Regulation of airway muscarinic cholinergic receptor subtypes by chronic anticholinergic treatment.. Mol Pharmacol.

[47] Liu Q, Wu J, Wang X, Zeng J (2007). Changes in muscarinic acetylcholine receptor expression in form deprivation myopia in guinea pigs.. Mol Vis.

